# Timing the Juvenile-Adult Neurohormonal Transition: Functions and Evolution

**DOI:** 10.3389/fendo.2020.602285

**Published:** 2021-02-12

**Authors:** Celia G. Barredo, Beatriz Gil-Marti, Derya Deveci, Nuria M. Romero, Francisco A. Martin

**Affiliations:** ^1^ Molecular Physiology of Behavior Laboratory, Department of Molecular, Cellular and Developmental Neurobiology, Cajal Institute, Spanish National Research Council (CSIC), Madrid, Spain; ^2^ Sartorius Netherlands BV, Amersfoor, Netherlands; ^3^ Developmental Timing, Environment and Behaviors Laboratory, Institut Sophia Agrobiotech, Université Côte d’Azur-INRAE-CNRS-INSERM, Sophia Antipolis, France

**Keywords:** metamorphosis, puberty, Urbilateria, Drosophila, sleep, juvenile-adult transition, neuro-hormonal regulation, neuroendocrine axis

## Abstract

Puberty and metamorphosis are two major developmental transitions linked to the reproductive maturation. In mammals and vertebrates, the central brain acts as a gatekeeper, timing the developmental transition through the activation of a neuroendocrine circuitry. In addition to reproduction, these neuroendocrine axes and the sustaining genetic network play additional roles in metabolism, sleep and behavior. Although neurohormonal axes regulating juvenile-adult transition have been classically considered the result of convergent evolution (i.e., analogous) between mammals and insects, recent findings challenge this idea, suggesting that at least some neuroendocrine circuits might be present in the common bilaterian ancestor Urbilateria. The initial signaling pathways that trigger the transition in different species appear to be of a single evolutionary origin and, consequently, many of the resulting functions are conserved with a few other molecular players being co-opted during evolution.

## Introduction

### Timing Metamorphosis as Compared to Puberty

**Figure 1:**
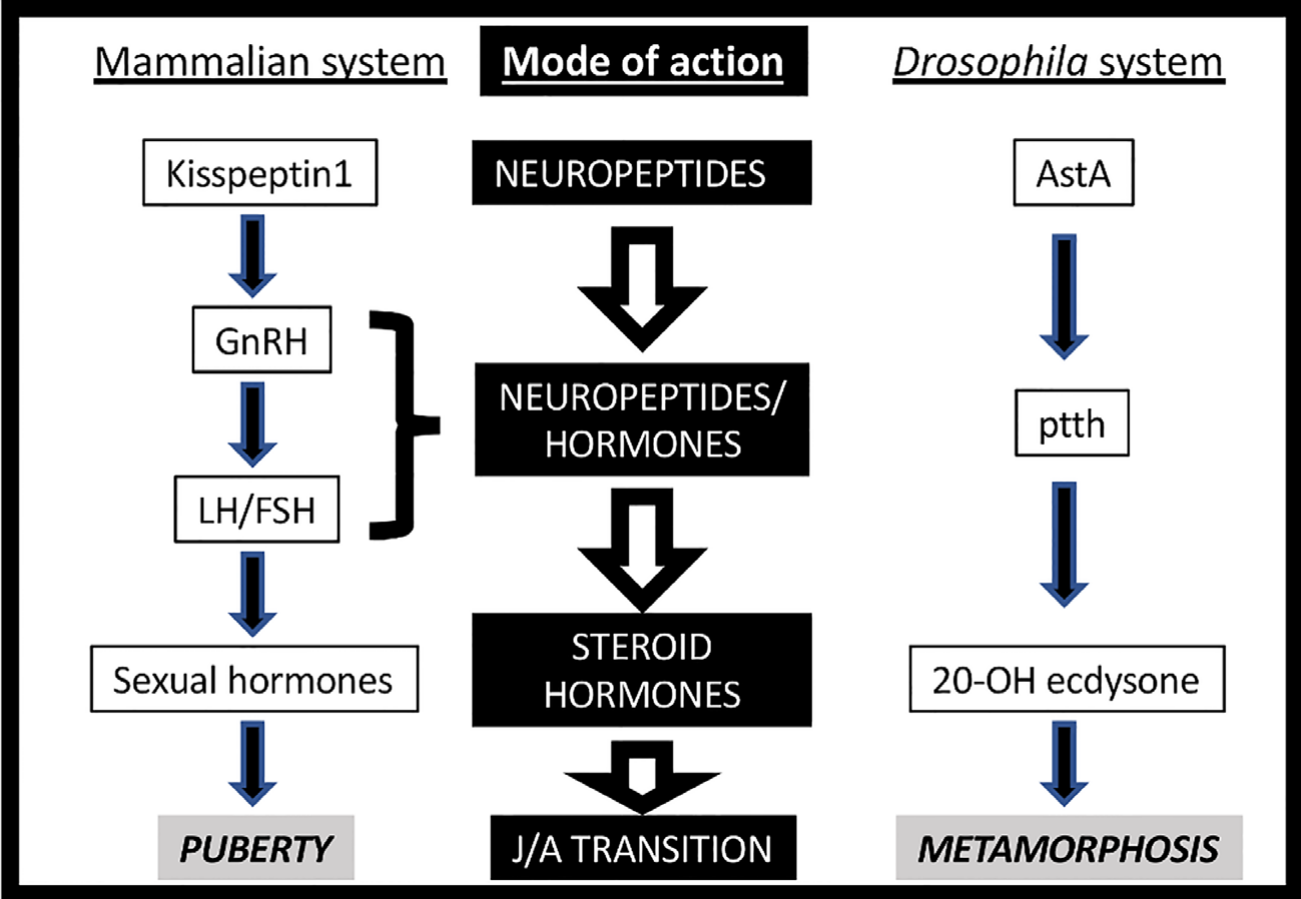
Neurohormonal axes triggering Juvenile-Adult (J/A) transition in mammals (left) and insects (right).

Life is based on the transmission of genetic material across generations. In multicellular animals, the most frequent (and consequently the most successful) strategy is through sexual reproduction. It usually requires a juvenile-adult transition in order to reach sexual maturation. Both mammals and insects trigger this process through a complex, multi-step neurohormonal signaling that may show certain structural parallelisms (**Figure 1**) (1, 2). Indeed, the neuroendocrine axis is activated at the pre-pubertal and pre-metamorphic stages by the activity of a group of neurons, which transmitted the initial stimulus to the mammalian pituitary gland or the *Drosophila* PG (prothoracic gland). These neurons, named GnRHn (Gonadotropin Release Hormone-expressing neurons) in mammals and PTTHn (Prothoracicotropic Hormone-expressing neurons) in insects, project their axons out of the brain barrier towards the ME (median eminence) or the PG to secrete the GnRH and PTTH neuropeptides, respectively. GnRH activates its GPCR (G-protein-coupled receptor) named GnRHR in the anterior pituitary cells inducing the circulating secretion of FSH (Follicle-stimulating Hormone) and LH (Luteinizing Hormone), which stimulates the production of steroid hormones (estrogens) in the gonads. Instead, PTTH activates its RTK (receptor tyrosine kinase) TORSO in the PG, arousing the MAPK/ERK (mitogen-activated protein kinases/extracellular signal-regulated kinases) signaling pathway, which leads to the direct stimulation of a steroid hormone (ecdysone) (3, 4). Moreover, the awakening of PTTHn and GnRHn activity at pre-metamorphic or pre-pubertal timing depends on the insect AstA/AstAR1 (AllatostatinA) and the mammalian Kiss1/Kiss1R (kisspeptin1) GPCR-dependent systems (**Figure 1**). The general scheme is very similar in terms of the design of the molecular players but not in the evolutive history, with the exception of AstA/kiss1 (see below). In addition to the hierarchical regulation of both axes, the different clusters of neurohormone-expressing cells also act as regulatory hubs (1, 2). For instance, GnRHn activity is controlled not only by kiss1 but also by other neuropeptides like neurokinin-B, dynorphin, NPY and the neurotransmitter GABA, among others (1).

The complexity of the mammalian neurohormonal axis (also known as hypothalamic–pituitary–gonadal -HPG- or reproductive axis) is reflected in the imbricated architecture of the cellular circuitry in contrast with the apparent simple beauty of insect one, with fewer cells and genes. Nevertheless, new players have been found recently that added complexity and solved an apparent paradox: whereas the PG down-regulation of ecdysteroidogenic enzymes arrests the developmental transition (as shown by the absence of pupariation), removing or silencing *ptth* or *torso* only creates a delay in pupariation onset. In the last decade, several studies have pointed out the PG as another node (in addition to the brain), which also integrates signals to regulates ecdysteroidogenesis. Indeed, Cruz and colleagues recently described that prothoracic EGFR (Epidermal Growth Factor Receptor) stimulated by VEIN and SPITZ ligands activate MAPK/ERK pathway in the PG, as PTTH/TORSO signaling also does (5, 6). EGF signaling might compensate for *ptth loss of function (LOF)*. *EGFR LOF* causes developmental arrest, resembling the lack of ecdysone (5). The autonomous PG expression of *split* and *vein* ligand genes depends on ecdysone signaling itself, suggesting that the EGFR pathway acts as an amplifying signal of the ecdysone production in the PG, a role previously described for ecdysone/EcR signaling (7, 8). Conversely, PTTHn are controlled by developmental and environmental inputs like growing tissue damages, photoperiod, over-crowding conditions, and ecdysone itself (2, 8). PTTHn also receive inputs from other neuronal circuits like the AstAn (9) and the corazonin (*crz*) neurons (10). Therefore, it is suggested that PTTH might be the link between the endocrine system and the internal/external milieu and other signals like EGF and ecdysone itself are required autonomously in the PG to ensure enough ecdysone surge to provoke metamorphosis (2).

Another enigma in the insect model comes from the difference in the observed developmental delay between the *ptth* null mutant (one day) and the PTTHn ablation (5 days). This suggests that PTTHn may cause electrical stimulation of the prothoracic gland to promote the release of ecdysone (11), or they may secrete another signal(s) that regulates ecdysone synthesis (12). Future studies are necessary to disclose the full PTTH neuronal function in timing the juvenile to adult transition.

## The Neurohormonal Axis During Juvenile to Adult Transition and Fertility

**Figure 2:**
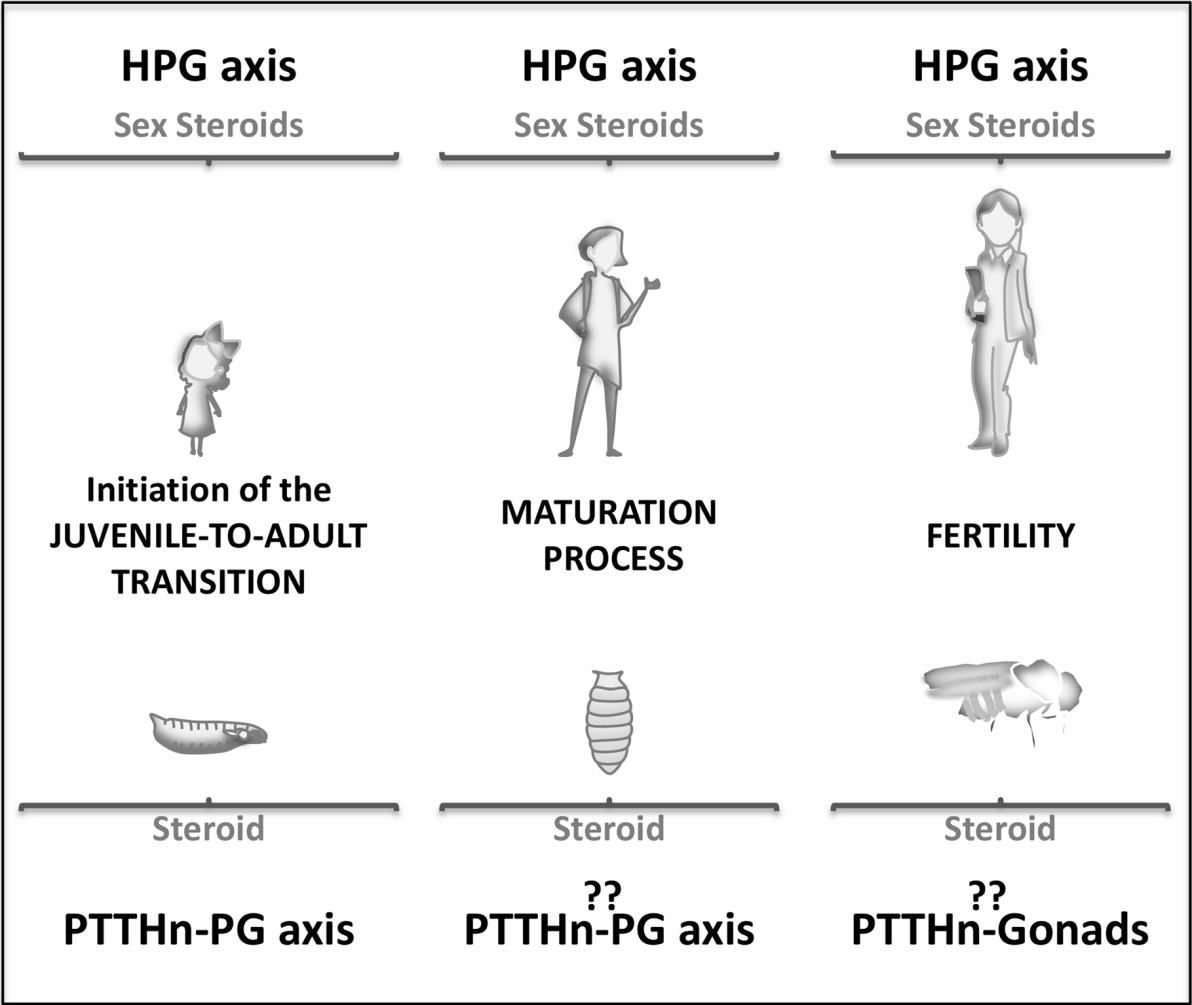
Schematic representation of the J/A neuroendocrine axis involvement during human (up) and Drosophila (down) lifespan. Juvenile stages (left), pubertal or metamorphic stage (center), and adult stage (right).

Previous to adolescence and near to birth, the mammalian neurohormonal axis is active for a relatively short post-natal period known as mini-puberty (13, 14). This initial increase in the levels of sexual steroids is important for the correct development of gonads. Whether something similar may also happen in insects involving early pulses of ecdysone needs further investigation, although in larval stages three small ecdysone peaks of unknown function and non-related to ecdysis are described (15) After triggering puberty, the mammalian hypophysiotropic axis controls the overall pubertal process (somatic and sexual maturation) through steroid hormones (**Figure 2**). In *Drosophila*, the transition is commanded by ecdysone through the metamorphosis process. However, the role of PTTH in stimulating ecdysteroidogenesis during metamorphosis is not clear. *ptth* null mutant animals do not show any defect on the metamorphic process (12) although *ptth* transcription is highest at this stage (20 times higher than at larval or adult stages) (16). This result implies either that metamorphic PTTH does not stimulate ecdysteroidogenesis or, alternatively, that the role of PTTH in stimulating ecdysone synthesis during metamorphosis is compensated when absent (**Figure 2**). Moreover, *crz* or *AstA* silencing does not disturb metamorphic process itself. Therefore, whereas *GnRH* is essential for the pubertal process since its deficiency resulted in infertility and improper development of gonads, lack of *AstA*, *crz*, and *ptth* does not disrupt metamorphosis (like ecdysone absence does). This suggests the existence of another intrinsic/regulatory mechanism of ecdysone production in insects. Indeed, some hemimetabolous and ametabolous insects lack *ptth*, and the involvement of CRZ in their metamorphic process has not been described (17–19). Further studies should better clarify the existence of other ecdysone regulatory/intrinsic mechanism that might question the relevance of *AstA*, *crz*, and *ptth* signaling in timing Juvenile-to adult transition. Hence, the role of the PTTH-PG axis in the metamorphosis process itself remains controversial. Nevertheless, there are pieces of evidence indicating that the PTTH-PG axis is present during metamorphosis and regulates the rhythmicity of eclosion (i.e., pupal emergence) (20). PTTH acts as an intermediate player between the central clock, PDF-positive neurons, and the PG to produce ecdysone in a coordinated and timely manner to induce such rhythmic eclosion (20).

What is the role of these neurohormonal axes and their neuropeptide players in the adult stage? Are they still expressed and functional? In mammals, the pulsatile pre-pubertal secretion of GnRH enables the final maturation of a GnRH surge, which triggers the first ovulation (**Figure 2**) (21). Subsequently, the HPG axis regulates the ovulatory cycle in the female adult. In *Drosophila*, the fertility of adult females is also controlled by the ecdysone produced at the ovaries since the prothoracic gland degenerates by the end of metamorphosis (22, 23). In the adult brain, PTTH expresses in a group of neurons, but during metamorphosis, the larval brain undergoes extensive remodeling, and many new neurons are added. In this rewiring scenario, no precise analyses have been done to determine whether or not PTTH adult neurons are conserved from the juvenile larval stage. Moreover, adult PTTHn does not show projections towards the ovaries to directly control ovary ecdysteroidogenesis and reproduction (3). These observations may imply that the adult neuroendocrine axis is not identical to the larval one. Future research is needed to determine whether PTTH control adult ecdysteroidogenesis and reproduction as the mammalian neuroendocrine axis does.

## Transitional Neurohormones in the Adult

In contrast with the reduced number of larval *AstA*-expressing neurons, adult *AstA* expresses in several neurons and endocrine cells (23). The existence of several *AstA*-producing cell populations suggests distinct AstA roles in adult physiology, including behavior. The most conserved and studied function of AstA/AstAR signaling during adulthood refers to feeding and hunger regulation (23, 24). The activity of AstA-expressing neurons provides a satiety signal and regulates systemic Insulin signaling. Lowering AstA levels in enteroendocrine secretory cells (EESs) from the gut induces early midgut senescence and shortens lifespan (24). In part, AstA mediates sugar-mediated satiety through the mushroom body, inhibiting dopaminergic input neurons that results in suppressed food search (25). This suppression of neuronal dopamine activity is essential to form long-term appetitive memory after sugar ingestion (26).

Sleep is also affected by AstA signaling in the Posterior Lateral Protocerebrum (PLP) cell cluster. These neurons are targets of the circadian PDF-expressing neurons, affecting sleep but not rhythmicity (23). AstA functionally links PLPs with an important sleep center such as the central complex through AstAR1, although the physical connection is still missing (27). Interestingly, input dopaminergic neurons of the mushroom body also play a role in sleep regulation, but a possible role of AstAn has not been explored yet (28). It is tempting to speculate that AstA acts as a single key molecule that can coordinate an energy-saving activity like a sleep state with general metabolic homeostasis regulation at several levels.

As discussed above, the insect AstA system is evolutionary related to the mammalian GAL and KISS1 system. The small neuropeptide GAL and its three described receptors are widely distributed in several neuronal populations of the central and peripheral nervous systems and in other tissues such as gastrointestinal tract (29). In contrast, *Kiss1* expression is more restricted to particular neuronal populations of the hypothalamus (rostral periventricular area -R3PV- and arcuate nucleus -ARH-) and the amygdala (1). KISS1n are classically related to reproduction; however, their axons project to many other targets than solely GnRHn. Concerning feeding and metabolism, controversial data propose that the activation of GAL signaling has a transiently (up to 24 h) positive effect on sugar feeding ameliorating insulin resistance (29). A recent work implies the lateral hypothalamic subset of GALn in regulation of food reward, however the implication of GAL signaling is not studied (30). KISS1n themselves control feeding behavior, albeit independently from KISS1 signaling (31). A KISS1-related effect on metabolism has been reported in *Kiss1R*-deficient aged (but not young) mice, which are obese without increased feeding (32–34). Therefore, GAL and KISS1 are somehow connected to feeding and metabolism regulation, but they do not play the essential role that the AstA system does.

By comparison, the link between GAL signaling and sleep is well established. Preoptic GAL neuronal activity and expression are essential for sleep rebound after sleep deprivation (35). These results indicate that GALn, by means of GAL, is the output of the sleep homeostat in vertebrates. KISS1 function itself is not related to sleep regulation, although Kiss1n from the arcuate nucleus do play a key role in the control of circadian rhythms such as sleep, temperature, locomotor activity, and food consumption, probably downstream of the central clock (36). The most extensively established functions of GAL signaling are anxiety/depression and addiction, which are often co-morbid with addictive behaviors (37). Increased GAL signaling is associated with depression-like states. GALR 1 and 3 mediate this effect by interacting with the Serotonin receptor whereas GALR2 plays an opposite antidepressant and anxiolytic role (38). This differential effect may explain why increased GAL activity induces alcohol consumption, but it protects against other drugs of abuse (such as nicotine and opiates) (38). KISS1 signaling also has an impact on anxiety-like behaviors, which may be *GnRH*/estrogen-mediated or exclusively Kiss1R-mediated in the hippocampus (39). KISS1 regulates reproduction-related behaviors such as male sexual preference mediated by *GnRH* neurons, although a copulatory behavior like lordosis (the arching of female back in response to male copulation) is controlled by Kiss1n independently of KISS1 (40). A possible relation of AstA pathway with anxiety-like or addiction behaviors remains to be studied in *Drosophila* models of such behaviors. The only suggestive data is that high levels of AstA induce aggressiveness in Africanized honeybees (41).

In the adult brain, ecdysone regulates circadian rhythmicity through Let7 (although it is not clear where ecdysone is produced) (42). *ptth mRNA* is also detected in adult heads, with levels that change following a 24-h cycle (42). It has been proposed that PTTH controls adult ecdysone production participating in a regulatory feedback loop of the circadian clock although down-regulating *ptth* receptor *torso* does not produce any rhythmic alterations in the locomotion activity (20, 42), a classical readout of circadian rhythm in some insect and mammalian models. According to a *ptth* reporter that reflects faithfully larval and pupal expression, *ptth* expression in the adult is restricted to the Ellipsoid Body, a part of the Central Complex associated with sleep regulation (3, 27). This expression pattern is compatible with a role for *ptth* in sleep, although this possibility remains unexplored until now. In adult stages, PTTH behavioral function requires further studies, as well as its role in biological rhythms and steroid production.

Conversely, the adult role of CRZ has been studied in much more detail in regards to stress signaling metabolism and reproduction-related behaviors (43). *crz* is expressed in the somata of neurosecretory cells, among others. Down-regulation of *crz* in these cells or *crzR* in the IPCs (Insulin Producing Cells) prolongs survival in flies exposed to starving conditions, and also affect lipid and glucose metabolism (44). The knockdown of *crzR* in salivary glands and fat body (functionally equivalent to mammalian fat cells and liver) causes similar metabolic phenotypes, including increased triacylglyceride levels and feeding, after exposure to different stress conditions (45). Furthermore, in the marine bristle worm *Platynereis dumerilii* CRZ signaling coordinates metabolic state with the lunar phase in order to achieve the final size and induce sexual maturation (46). Intriguingly, *crz*- and *crzR*- expressing neurons in the abdominal ganglia regulate sperm transfer and copulation duration in males (47). The activation of CRZ signaling is sufficient to mimic ejaculation-caused reward, driving appetitive memories and reducing ethanol consumption (48). It was previously shown that CRZ regulates ethanol sensitivity and detoxification (43).

The presence of many GnRH neurons outside the hypothalamus in transparentized human fetuses, and the expression of GnRHR in several adult brain structures (which include the cortex, spinal cord, cerebellum, and hippocampus) suggest that GnRH signaling plays additional roles even after adolescence (49, 50). Most of them depend on its estrogenic activity, like fertility. Low GnRH levels and high GnIH (Gonadotropin Inhibitory Hormone, the main GnRH antagonist), correlate with the severity of human insomnia, probably through circadian estrogens regulation (51, 52). The role of estrogens in sleep and metabolism has been extensively studied and is described elsewhere (53). In addition, given the increasing evidence that GnRH signaling has neurotrophic, neuroprotective, and regenerative functions, it has been proposed that combined therapy of GnRH with Growth Hormone may be beneficial following neural damage (50). For instance, GnRH signaling in the female hippocampus regulates the synaptic plasticity through an estrogen-mediated mechanism (54).

## Comparison of Neurohormonal Axes and Their Evolutionary Origin

One controversial issue is how similar the overall design of GnRHn and PTTHn neuroendocrine hubs is since both trigger very similar processes, puberty and metamorphosis, respectively. In humans, GnRHn unmyelinated projections have unipolar or bipolar morphology that functions as a dendrite and axon simultaneously, a structure named dendron (55). These projections travel to the ME of the pituitary gland covering distances over 1–3 mm (56). Once in the ME, GnRHn projections branch into multiple short axons with “specialized” neuroterminals boutons that target the blood vessels from the closed portal vasculature. There, GnRH is released to act on the anterior pituitary gland (57). In *Drosophila*, PTTHn projections also have a very simple unipolar morphology that functions simultaneously as a dendrite and axon and travels a long distance of up to 500 µm to reach the PG (3, 58). PTTHn projections branch into multiple short axons with “specialized” terminal boutons that target individual PG cells (12). GnRHn exhibit pulse and surge modes of activity to control fertility. Whereas the LH surge requires functional soma-proximal dendrites and distal dendron, the distal dendron integrates synaptic information to drive pulsatile GnRH secretion (59). Recent studies indicate the presence of regulatory mechanisms controlling GnRHn and PTTHn projections into the ME/PG, respectively (9, 60, 61). Actually, various genes involved in the Semaphorin signaling, like *semaphorin-3A* (*Sema3A*), *neuropilin* (*Nrp1* or *Nrp2*) coreceptors, and its receptor *plexin*-*A1* (*PlxnA1*), have been implicated in the guidance of GnRH neuronal migration, its survival and adult GnRHn plasticity affecting fertility (60, 62–66). Whereas PTTH neuronal development and migration towards the PG needs to be elucidated, a recent study suggests that PTTH neurons are also suffering from axon remodeling during the juvenile period (9). *Drosophila* Semaphorin signaling is well conserved, and it has also been implicated in axon guidance and synaptic plasticity (67). Interestingly, silencing the homologous of *PlxnA1* (*pLexB* receptor) in the PG caused a delayed onset of metamorphosis, suggesting that a similar mechanism regulates PTTH neuronal growth (68). Further studies should investigate the involvement of *Drosophila* Semaphorin signaling pathway in PTTH neuroterminal remodeling, migration towards the PG or survival, expanding or not the similarities between PTTH and GnRH neuronal development and physiology.

Unlike *Drosophila* PTTHn, which directly innervate the prothoracic gland itself, Lepidoptera PTTHn release PTTH into the hemolymph from their specialized nerve endings in the *corpus allatum* (CA) (69). The CA is the endocrine organ that produces Juvenile hormone (JH), the primary negative regulator of PTTH in the Lepidoptera Manduca sexta. JH drops upon the organism attainment of a critical weight allowing PTTH titters to rise and induce metamorphosis (70). In *Drosophila*, the function of JH on ecdysone synthesis has been the center of controversies. Indeed, removal of total JH by CA ablation or the use of different null mutants does not advance metamorphosis as expected by releasing the potential JH inhibitory effect on ecdysteroidogenesis (71). However, the silencing of the JH receptor exclusively in the PG induces ecdysone biosynthesis and triggers the precocious initiation of pupariation (72, 73). It has been proposed that this difference reflects a secondary effect of JH in the Insulin-mediated growth pathway, which antagonizes the direct timing effect on the PG. Further studies are needed to better clarify this point. Differences in the neuroendocrine connectivity between *Drosophila* and *Manduca* might reflect species-specific variances in ecdysone regulation. Indeed, in other insects like *Bombyx* mori, PTTHn also innervates the CA, raising the possibility that PTTHn projections towards the CA is a general trait in insects (74). Furthermore, *Drosophila* PTTH functions as a neuropeptide on the PG, whereas *Lepidoptera* PTTH acts as a circulating hormone (3, 12, 69). Indeed, PTTH in *Manduca sexta* and *Bombyx mori* shows more similarities with the mammalian GnRH, which does not directly reach the hypophysis and acts as a secreted hormone into the circulating portal vessels.

**Figure 3:**
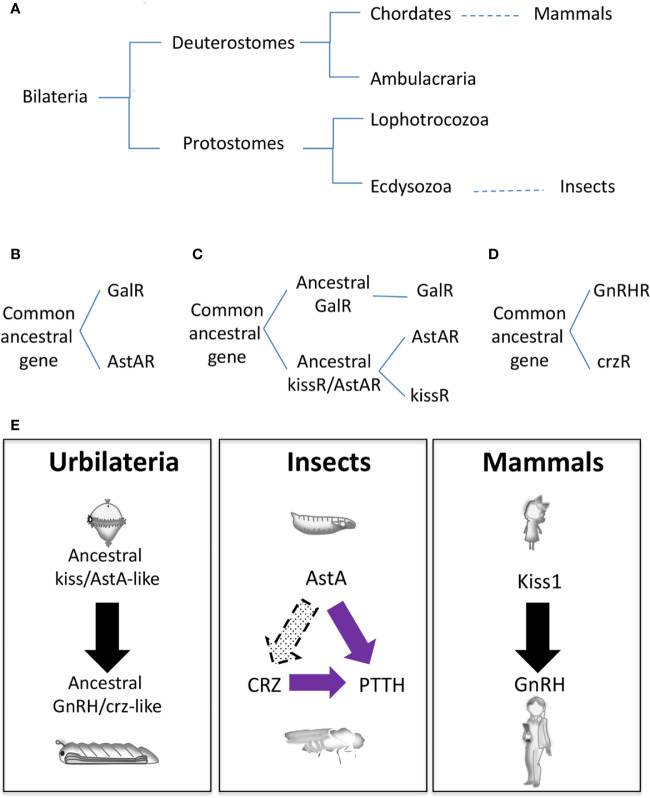
Evolutionary origins of the neuroendocrine gene network. **(A)** Animal phylogenetic diagram showing the position of mammals and insects. **(B–D)** Schematic representation of different hypothetical gene evolution for GalR, AstAR, kissR, GnRHR, and crzR. **(E)** Speculative neuroendocrine axis controlling J/A transition of Urbilateria (left) that has been evolutionary conserved in insects (center) and mammals (left).

Another pending question in the field is whether or not genes involved in the mammalian and insect neuroendocrine circuitry are evolutionary related, given the evolutionary distance (**Figure 3A**). Several phylogeny studies propose that the insect AstA system is evolutionary related to the mammalian GAL and KISS1, the mammalian gatekeeper of puberty (75–79). Indeed, *gal* and *kiss* emerged from the same ancestral gene precursor, and most likely this divergence occurs before protostome and deuterostome separation since *kissR* sequences have been found in protostomes such as annelids and mollusks (**Figure 3A**) **(** 78–80). Based on neuropeptide receptor analysis, the AstA system was initially evolutionarily associated with GAL (**Figure 3B**) (75, 76). Functional studies in the *Drosophila* AstA system support this evolutionary relation since AstA participates in similar biological processes as GAL (sleep, food intake, metabolism, gut physiology, and others; see above). Nevertheless, more recent phylogeny and gene synteny studies on the receptors and mature peptides might indicate that AstAR and KISS1R emerged after GALR gene divergence from the common ancestral gene (**Figure 3C**) (77, 78). This model is also reinforced by our finding that AstA regulates the juvenile-to-adult transition, as Kiss1 does in mammals (9). Moreover, KISS signaling in the non-chordate deuterostome sea cucumber *Apostichopus japonicus* regulates both reproductive and non-reproductive (glucose metabolism) functions as the AstA system does in *Drosophila*, hinting to a possible dual ancestral role for KISS/AstA signaling (81). These data favor the hypothesis that *AstA* may be the true *kiss1* orthologue while *gal* is a *kiss1* paralog gene. Further studies are required to determine the precise mammalian *AstA* orthologous and paralogous conclusively.

Whereas *AstA* and *Kiss1* are evolutionarily related, *GnRH* and *ptth* are non-homologous genes. PTTH, a cysteine knot protein, was the first hormone/neuropeptide identified in insects (82). It is part of the noggin/noggin-like family, an extracellular regulator of BMP (Bone Morphogenetic Protein) signaling first characterized in vertebrates (2). However, *ptth* has evolved from a *noggin-like* gene exclusively in insects being a common component of holometabolous, with no conservation in other insect species or in vertebrates (19). On the other hand, the PTTH receptor Torso (4) is an RTK, similar to the large numbers of RTKs present in animal genomes, making difficult its precise identification for evolutionary studies. However, some phylogenetic relationship analyses determine that *torso* appeared before the divergence of lophotrochozoans and ecdysozoans but not after the divergence of deuterostomes and protostomes (**Figure 3A**) **(** 19).

GnRH neuropeptide and its receptor GnRHR do exist in *Arthropoda*, which include insects. Evolutionary analyses suggest that a common ancestral gene in the *Bilateria* family gave rise to *GnRHR* and *crzR* (*corazonin receptor*) through gene duplication; after that *crzR* was lost in the vertebrates (**Figure 3D**) (83, 84). The second gene duplication in *Arthropoda* from *GnRHR* gave rise to *Adipokinetic Hormone* (*AKHR*) and *AKH/CRZ-related peptide Receptors* (*ACPR*), the latter being lost in *Drosophila* (not shown). Functional experiments uncovered an essential role of AKH in energy/metabolism homeostasis, acting as the equivalent of the mammalian glucagon, and providing evidence that AKH does not function as the ortholog of GnRH (85). Accordingly, circuitry analyses in *Drosophila* demonstrated that AKH positive neurons do not show a direct innervation to the prothoracic gland as PTTH neurons do or as GnRH neurons connect with the pituitary gland (86). Interestingly, *crz* is expressed in a group of neurons (CRZn) that project towards both the PTTHn and PG. Early in development *crzR* is expressed and functional in PTTHn and not in the PG, thus regulating systemic growth PTTHn-mediated but not the metamorphosis timing (10). These observations may indicate that the CRZ-PTTH circuit is more likely a recent innovation in insects. Alternatively, a cross-talk between CRZ neurons and AstAn may exist, which would suggest a more persuasive homology between the mammalian and insect neuroendocrine systems than previously thought (10).

It is tempting to speculate that an ancestral common neurohormonal axis regulating metamorphosis existed in *Urbilateria* (the putative common ancestor of protostomes and deuterostomes), which would probably have an initial ciliated free-swimming (pelagic) larval phase (**Figure 3E**) (87). This axis would diverge to mammalian KISS1/GnRH and insect AstA/CRZ. Not surprisingly, non-chordate deuterostomes like sea cucumber and starfish have functional KISS and GnRH signaling, respectively (81, 88), and the metamorphosis of an ancient chordate like the Ascidian *Ciona* is triggered by GABA-induced GnRH release (89). In this scenario, PTTH/TORSO signaling might have been co-opted later on during insect evolution and somehow substituted the transitional developmental function of CRZ (**Figure 3E**). In contrast, CRZ kept its role in systemic growth and food intake during starvation-induced stress response (67). Supporting this hypothesis, in the *diptera*-like Oriental fruit fly (*Batrocera dorsalis*) CRZ signaling regulates juvenile-adult transition, although *ptth/torso* genes are also present in the genome with no discernable role on pupariation (90, 91). Also, the initiation of *Manduca* ecdysis depends on CRZ activity, and the role of *ptth* on Manduca pupariation is firmly established (82, 92). In summary, we propose that a functional neuroendocrine axis comprising ancestral KISS/GnRH-like signaling, which regulated juvenile-adult transition and probably also stress-related metabolism, existed in *Urbilateria* (**Figure 3E**). Despite posterior modifications in other functions and the incorporation of new genes, this surprising conservation among phyla would mean that separating immature juveniles and sexually mature adults might be a successful and most likely unique evolutionary event. It will be of interest to explore this hypothesis further.

## Conclusions and Future Perspectives

The genetic network involved in the developmental switch display several functions in adulthood. While puberty and metamorphosis present common features regarding the central role of the brain controlling steroid production to initiate the developmental transition, the neuroendocrine axis looked simpler in insects than in mammals. The central neurons controlling gland hormone production involves GnRH and PTTH signaling pathways, with no sequence homology between *GnRH/GnRHR* and *ptth/torso* genes. These observations would suggest that both neurohormonal axes are just functionally analogous. However, we recently found that *AstA* is the *Drosophila* homolog for *kisspeptin1*, thus challenging this simple view. Moreover, a recent report on *Drosophila crz* together with novel evolutionary aspects of *kiss-GnRH* systems indicated that puberty and metamorphosis might be homologous processes. These data open the question of whether the neuroendocrine axis represents an evolutionary convergence or an evolutionary conserved mechanism timing Juvenile-to-adult transition between mammals and insects. Even if there are clear similarities between pubertal and metamorphic mechanism, several differences are also present.

In mammals, the adult HPG axis control steroid production to regulate fertility. In *Drosophila*, whereas it is not clear whether or not PTTH or CRZ regulate steroid production, steroid (ecdysone) does control adult fertility. The neuroendocrine gene network has also been found to regulate adult physiology and behavior both in mammals and *Drosophila*. GAL/KISS and AstA are involved in feeding/metabolism and sleep regulation, although with differential relevance. This may indicate not only the maintenance of putative ancestral metabolic roles but also the acquisition of novel functions. So the question is if we can model any dysregulations of the neuroendocrine axis or associated complex human pathologies (such as anxiety and addiction) in a simpler model system like *Drosophila*.

Future research might lead to a better understanding of the endocrine network evolution and confirm (or refute) the common origin of the neuroendocrine axis that controls the developmental transition to adulthood. Distinguishing similarities and differences in the juvenile to adult transition between mammals and invertebrates is an essential step in the process of using model organism to allows the identification of target elements to assay potential remedies to multifactorial ailments.

## Author Contributions

CB: writing – original draft. BG-M: writing – original draft. DD: writing – original draft. NR: conceptualization, funding acquisition, project administration, writing – original draft. FM: conceptualization, funding acquisition, project administration, writing – original draft. All authors contributed to the article and approved the submitted version.

## Funding

This work was supported by MICINN (Grant number PGC2018-094630-B-I00 to FM), Comunidad de Madrid (CB was a recipient of “Ayuda de Garantía Juvenil para la contratación de investigadores predoctorales” fellowship, grant number PEJD-2019-PRE/BMD-15940), CSIC (BG-M was a recipient of a JAE intro fellowship, grant number JAEINT_19_00602), Inserm (ATIP-Avenir program to NR) and the French National Research Agency -ANR- (“Investments for the Future” programs LABEX SIGNALIFE ANR-11-LABX-0028 and IDEX UCAJedi ANR-15-IDEX-01 to NR). FM is a recipient of a RyC-2014-14961 contract. MEFP (CGB is a recipient of a FPU predoctoral fellowship, grant number FPU19/04449), UAM (BG-M is a recipient of a predoctoral fellowship, grant number SFPI/2020/00878).

## Conflict of Interest

DD was employed by company Sartorius Netherlands BV.

The remaining authors declare that the research was conducted in the absence of any commercial or financial relationships that could be construed as a potential conflict of interest.
